# Investigating the maximum bite force and speech intelligibility in patients requiring prosthetic rehabilitation

**DOI:** 10.1111/jopr.70156

**Published:** 2026-05-04

**Authors:** Aditi Gupta, Nupur Patel, Francis Mante, Jason D. Lee, M. Brian Chang

**Affiliations:** ^1^ Division of Restorative Dentistry Department of Preventive and Restorative Sciences University of Pennsylvania School of Dental Medicine Philadelphia Pennsylvania USA; ^2^ Private Practice Claremont New Hampshire USA; ^3^ Department of Restorative Dentistry and Biomaterials Sciences Harvard School of Dental Medicine Boston Massachusetts USA; ^4^ Department of Oral and Maxillofacial Surgery University of Pennsylvania School of Dental Medicine Philadelphia Pennsylvania USA

**Keywords:** complete removable dental prosthesis (CRDP), edentulous patients, hybrid prosthesis, implant‐supported fixed dental prosthesis (IFDP), implant‐retained overdentures (IOD), masticatory function, maximum bite force, natural dentition, prosthodontic rehabilitation, speech intelligibility

## Abstract

**Purpose:**

Tooth loss leads to reduced occlusal contact area, altered jaw biomechanics, and diminished neuromuscular coordination, impairing both masticatory function and speech clarity. Edentulous patients often adapt by modifying food choices or swallowing behavior and may experience persistent phonetic disturbances. Maximum bite force affects masticatory performance, while speech intelligibility affects patient communication. This study aimed to objectively evaluate maximum bite force and speech intelligence among completely edentulous patients rehabilitated with different types of prostheses, compared with natural dentition.

**Materials and Methods:**

A cross‐sectional study (IRB #857207) was conducted with 50 participants divided into 5 groups: (A) implant‐supported fixed dental prosthesis (IFDP); (B) implant‐retained complete removable overdentures (IOD) in both arches; (C) maxillary complete removable dental prosthesis (CRDP) with mandibular IOD; (D) maxillary and mandibular CRDP; and (E) natural dentition (control). Maximum bite force was measured using a microelectromechanical (MEM) pressure sensor during three maximal‐effort bites; unilateral values were also analyzed. Speech intelligence was assessed using a standardized articulation passage and analyzed with a speech intelligibility scorer; readings were taken three times per participant.

**Results:**

Group E had the highest masticatory bite force (752.03 N), followed by Groups A (522.97N), B (294.13 N), C (174.23 N), and D (72.13 N). Total bite force was 1.5–1.8× greater than unilateral values. Group E also had the highest speech score (85.67%) and outperformed Groups B, C, and D (*p* < 0.005). Fixed prostheses and natural dentition (Groups A and E) outperformed removable designs (Groups B, C, and D) in both maximum bite force and speech intelligibility. Males had higher bite force than females (*p* < 0.01); no statistically significant gender differences were found for speech.

**Conclusion:**

This study underscores the need for individualized assessment strategies in prosthodontic rehabilitation by establishing an objective functional gradient across prosthetic designs. Complete removable dentures exhibited only one‐tenth of the maximum bite force of natural dentition. Maximum bite force improved to 2.4× with a mandibular IOD and 4× with IOD in both arches. Fixed prostheses yielded superior speech intelligibility due to enhanced tongue space and palatal contours. Total bite force was consistently greater than unilateral values. While gender influenced bite force, it did not affect speech. Bite force sensors and intelligibility scorers offer efficient, evidence‐based tools for long‐term evaluation.

Maximum bite force (mBF) is a well‐established measure of the functional capacity of the masticatory system and decreases with tooth loss, with factors such as age and gender further influencing outcomes.^1^ Miyaura et al. demonstrated that tooth loss reduces occlusal contact area and alters biting patterns.^2^ As early as 1950, Manly and Braley noted that edentulous patients adapt by swallowing larger particles or avoiding certain foods altogether.^3^

Beyond its clinical relevance, mBF reflects the action of jaw elevators and the biomechanics of craniofacial musculature.^4^ Koshino et al. emphasized the importance of objective mBF assessment in prosthodontics, highlighting its relationship with eating satisfaction, comfort, nutrition, and overall patient well‐being.^5^ Thus, mBF is not only a surrogate for masticatory function but also a predictor of prosthodontic treatment success.^6^ Physiologic bite force depends on a combination of factors, including dentition status, age, gender, muscle condition, and facial morphology.^7^

Because of this, measurement of mBF is a tool used in dentistry to evaluate the effectiveness of prosthetic devices.^8^ Miyaura et al. compared mBF among patients with complete removable dental prosthesis (CRDPs), fixed partial dentures (FPDs), removable partial dentures (RPDs), and natural dentition, showing that biting forces were only 80%, 35%, and 11% of natural dentition values for FPD, RPD, and CRDP groups, respectively.^2^ Similarly, Lasilla et al. demonstrated that maximum bite force differs between dentate, edentulous, and partially edentulous patients, with correlations to occlusal stability and biting ability.^7^ Fontijn‐Tekamp et al. further showed that implant‐retained overdentures produced significantly higher forces than conventional dentures, though still lower than dentate subjects, regardless of recording site (canine, premolar, or incisor).^9^ Collectively, these studies underscore the influence of prosthetic design on masticatory performance, making awareness of such differences essential for treatment planning.

Several methods have been developed to record mBF. Borelli's earliest attempt in 1681 used weights attached to mandibular teeth,^10^ later replaced by micrometer‐based devices and, ultimately, electronic instruments.^11^ More recently, microelectromechanical (MEM) pressure sensors—Innobyte have been introduced, offering ISO 17025–calibrated precision (1% variation, 5% total error across a 2000 N range) and clinical applicability.^12^

Despite extensive investigation of maximum bite force within isolated prosthetic modalities, objective comparisons of mBF across contemporary fixed and removable prosthodontic treatments using chairside instrumentation remain limited.

Phonetics has long been integrated into prosthodontic treatment as a guide for denture design. In 1962, Sharry described its application as more of an “art” than a precise science. Since then, structured phonetic tests have been introduced for complete denture fabrication.^13^ Additional phonetic cues, including sibilants like “s” and “z,” have been used to assess palatal thickness or vertical dimension.^14^ Despite these techniques, speech problems remain frequent in patients with fixed prostheses. Lundqvist et al. reported that 60% of patients exhibited distorted speech immediately after treatment. Thirty percent continued to do so after 3 years, and after 9 years, errors remained in 82% of implant prosthesis wearers compared with 52% of dentate subjects.^15^ The mucosa–prosthesis gap in fixed prosthetics and the palatal coverage of removable appliances both contribute to these errors, leading modern prosthodontics to incorporate tools such as palatography and phonetic assessment for both removable dentures and complete‐arch fixed implant prostheses.^16^, ^17^

Conventional methods of speech evaluation rely on subjective assessment. Knipher introduced an objective, software‐based approach that quantifies word accuracy through standardized speech samples, yielding a reproducible “speech intelligibility score.”^18^ Yet such approaches have not gained widespread recognition as reliable parameters for evaluating prosthetic outcomes, and most assessments remain limited to fabrication steps or immediate evaluation. Although phonetic principles are routinely incorporated into prosthodontic fabrication and speech is commonly evaluated during treatment, current prosthodontic literature lacks an objective, chairside assessment of speech intelligibility (SI) across prosthetic designs, particularly for post‐treatment functional evaluation. This article introduces SI as an objective method to assess speech production after prosthesis delivery.

This study, therefore, aimed to evaluate mBF and SI in completely edentulous patients rehabilitated with different prosthodontic treatments, using standardized chairside tools, and to compare these outcomes with natural dentition. The null hypothesis was that there is no statistically significant difference in mBF or SI across all prosthetic groups versus natural dentition.

## MATERIALS AND METHODS

This cross‐sectional study included 50 participants divided into 5 groups (*n* = 10 each):

- Implant‐supported fixed dental prosthesis (IFDP): At least one arch restored with a full‐arch, implant‐supported fixed complete denture opposing the same or natural dentition, while preserving natural palatal morphology, patients had varied intaglio surface designs in accordance with available inter‐arch space.
- Implant‐retained complete removable overdentures (IOD, both arches): Completely edentulous arches restored with two to four implant‐retained overdentures with locator attachments. Maxillary IODs were designed with a horseshoe design with a palatal plate fabricated in metal.
- Maxillary CRDP + mandibular IOD: Maxillary complete denture and mandibular two‐implant overdenture with locator attachments.
- Maxillary and mandibular CRDP: Conventional complete removable dental prosthesis in both arches.
- Natural dentition (control): Healthy dentition (28–32 teeth, with or without third molars), no malocclusion, equal male and female representation.

Power analysis was performed with SigmaStat software, referencing Knipher's study for SI (SD = 7.5) and Rismanchian's study for maximum bite force (SD = 1.6). The required sample size was determined to be 10 per group. The study was approved by the institutional review board (protocol #857207), and written informed consent was obtained from all participants in accordance with 45 CFR 46.110.

Inclusion criteria were patients who had completed prosthodontic treatment for edentulous arches representative of the treatment groups, adults with natural dentition, and patients who provided consent to participate in the study; exclusion criteria included craniofacial defects, neurological deficits, hearing impairment, or inability to speak English. Patient data were collected through interviews, examination, and chart review from treatment performed at the university.

mBF was recorded with Innobyte (Kube Innovation). It is a MEM pressure sensor device, chosen for its clinical applicability based on a study by Ustrell‐Barral et al. that reported a mean error of ∼25 N (<4% of natural dentition mBF) with this system. The device employs micro‐scale structures that detect membrane deflection, converting it into electrical signals.^12^

Participants were seated upright with head support, and a disposable bite element was placed between the first molars. They performed three maximal 3‐s bites per side with 60‐s rests; the average values were analyzed for bilateral and unilateral forces, with the higher unilateral value designated as dominant bite force.

Speech was evaluated using a standardized articulation passage (Table 1),^19^ designed to reflect English phonemic distribution, incorporate all major consonant sounds, and minimize random error within a 45–60‐s reading. Participants read the passage in English into a microphone connected to a laptop running the SI scorer by icSpeech (Rose Medical Solutions), which provides real‐time visualization and calculates an intelligibility score (0%–100%). Recognized words were highlighted in green; unrecognized words were deducted from the score.

**TABLE 1:** Kestenberg's speech assessment test.

How are you Tom Dope? No oranges are growing in Mexico. It is nice to see my grandfather swim about here. George depends on Ruth to bake a big lemon cake. Roses are red and Violets are blue. Tim, show Harry where to wash your clothes tub. Wednesday will be a laugh for all of us. The sixty‐five fast trucks leave the zoo each year. Perhaps you need to fire the man in England too. Sweet Peggy Nun caught that fur hat. The children weren't catching anything.

*Note*: This passage is a standardized articulation test containing a representative distribution of English phonemes, used for objective speech intelligibility analysis.

The software was configured with the following parameters: restricted vocabulary (text only), auto‐skip unrecognized words, rejection threshold set at zero, and search for up to 100 lexical alternatives.^20^ Each recording was repeated three times per participant, with the average score used for analysis. Although widely applied in speech pathology, this tool has not previously been reported in denture phonetics.

### Statistical analysis

Data were analyzed using SigmaStat (Systat Software). Mean and standard deviation were calculated for each group. Normality was verified (Shapiro‐Wilk; Box‐Cox transformation as needed). Two‐way ANOVA tested differences in mBF and SI across prosthesis type and gender, with pairwise comparisons by the Holm‐Šidák method. For SI, Groups A and E were combined as fixed prostheses, and Groups B–D as removable prostheses.

Relative ratios were derived from least squares means and reported only when post hoc tests confirmed significance (*p* < 0.05). The Pearson correlation coefficient evaluated the association between dominant and nondominant mBF. Repeated‐measures ANOVA with Bonferroni‐corrected *t*‐tests compared total, dominant, and nondominant bite force, with ratios calculated to quantify differences.

## RESULTS

The study cohort comprised 50 participants distributed across 5 prosthetic groups. In Group A (IFDP, *n* = 10), patients (age range: 35–76 years; 6 males, 4 females) were rehabilitated with implant‐supported fixed dental prostheses utilizing 4–6 implants per arch, with mixed maxillary and mandibular fixed reconstructions and preservation of natural dentition in the opposing arch in selected (3) cases. Group B (IOD in both arches, *n* = 10) included patients aged 45–80 years (6 males, 4 females) restored with implant‐retained overdentures supported by 2–4 implants in the mandibular arch and 4 implants in the maxillary arch. Group C (maxillary CRDP with mandibular IOD, *n* = 10) consisted of patients aged 56–79 years (5 males, 5 females) rehabilitated with a maxillary complete removable dental prosthesis and a mandibular implant‐retained overdenture supported by 2 implants. Group D (CRDP, *n* = 10) included fully edentulous patients aged 38–75 years (2 males, 8 females) treated with conventional complete removable dental prosthesis in both arches. The control group, Group E (natural dentition, *n* = 10), comprised dentate individuals aged 26–33 years (5 males, 5 females). It was not age‐matched to other groups, as it was selected to represent an age range associated with peak maximal bite force in adults prior to age‐related decline.^21^ Prostheses were completed between 2019 and 2025, with all participants enrolled and evaluated during the 2025 study period.

Table 2 summarizes descriptive statistics (mean, SD, least squares mean, SEM) for mBF and SI across different prosthetic groups.

**TABLE 2:** Maximum bite force measurements, speech intelligibility scores across groups, and speech intelligibility scores for fixed and removable prostheses.

Bite force measurement across prosthetic groups				
**Group**	**Mean (N)**	**SD (N)**	**Least squares mean (N)**	**SEM (N)**
A—IFDP	535.1	164.41	522.97	26.57
B—IOD (both arches)	297.27	152.61	294.13	25.81
C—Maxillary CRDP + mandibular IOD	174.23	92.05	174.23	26.03
D—CRDP	71.03	41.91	72.13	32.54
E—Natural dentition	752.03	264.52	752.03	26.03

**Speech intelligibility score across prosthetic groups**				
**Group**	**Mean (%)**	**SD (%)**	**Least squares mean (%)**	**SEM (%)**
A—IFDP	70.13	17.37	70.13	3.17
B—IOD (both arches)	56.52	18.22	56.52	3.36
C—Maxillary CRDP + mandibular IOD	58.17	18.27	58.17	3.34
D—CRDP	49.96	20.91	49.96	4.18
E—Natural dentition	85.67	8.93	85.67	1.63

**Speech intelligibility score for fixed (X) and removable prosthesis (Y)**				
**Group**	**Mean (%)**	**SD (%)**	**Least squares mean (%)**	**SEM (%)**
X—Fixed prosthesis	77.91	15.77	77.94	2.04
Y—Removable prosthesis	54.90	16.70	54.90	1.77

*Note*: Values are expressed as mean, standard deviation (SD), least squares mean (LSM), and standard error of the mean (SEM).

Abbreviations: CRDP, complete removable dental prosthesis ; IFDP, implant‐supported fixed dental prosthesis; IOD, implant‐retained overdenture.

**FIGURE 1:**
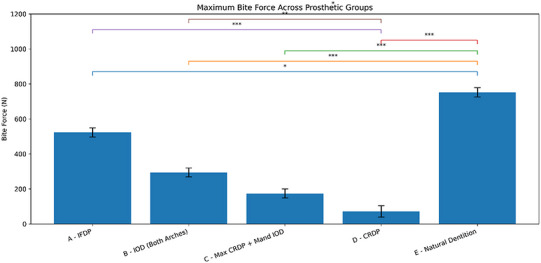
Maximum bite force (mBF) across prosthetic groups. Bars represent least squares mean (±SEM) values for each group: A—implant‐supported fixed dental prosthesis (IFDP); B—implant‐retained overdentures (IOD, both arches); C—maxillary CRDP + mandibular IOD; D—complete removable dentures (CRDP); E—natural dentition. Statistical significance is indicated as follows: *p < 0.05, **p < 0.01, ***p < 0.001.

Figure 1 shows that the analysis of mBF across different dental and prosthetic treatments is evident. The control group (natural dentition, Group E) exhibited the highest bite force (*p* < 0.001). Among prosthetic treatments, the IFDP group (Group A) demonstrated the highest bite force, with significantly higher bite force than groups using CRDP and IOD (*p* < 0.01).

mBF was significantly lower in CRDP wearers (Group D) compared to all other groups. Groups using IODs in both arches (Group B) and maxillary CRDP with mandibular IOD (Group C) showed moderate mBF but were significantly outperformed by the IFDP and natural dentition groups.

A gradient in mBF was observed, with natural dentition > IFDP > IOD > maxillary CRDP + mandibular IOD > CRDP.

**FIGURE 2:**
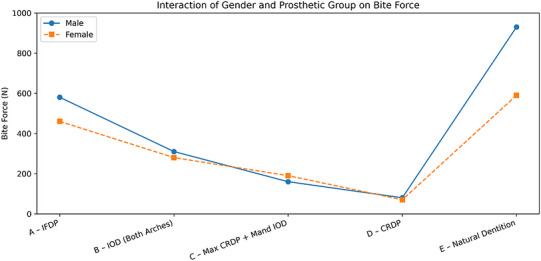
Interaction plot of gender and prosthetic group on maximum bite force (mBF). Least squares mean values (±SEM) are shown for males and females within each prosthetic group. CRDP, complete removable dental prosthesis; IFDP, implant‐supported fixed dental prosthesis; IOD, implant‐retained overdenture.

Males exhibited significantly higher mBF (408.38 N) than females (317.28 N) (*p* < 0.01). Figure 2 presents an interaction plot of prosthetic group by gender, showing the least squares mean mBF (±SEM) for males and females across all groups. In all groups, males generally exhibited higher mBF than females. The most pronounced gender difference was observed in Group E (natural dentition), where males (930.47 ± 42.72 N) far exceeded females (582.13 ± 38.92 N). A smaller but statistically significant (*p* < 0.05) difference was also observed in Group A (IFDP), with males (578.13 ± 34.63 N) showing higher values than females (460.24 ± 31.71 N). In contrast, Groups B (IOD), C (Max CRDP +Mand IOD), and D (CRDP) showed no significant gender‐related differences, with means of 310.40 ± 28.21 versus 285.58 ± 30.48 N, 159.27 ± 26.09 versus 188.63 ± 27.44 N, and 74.50 ± 18.18 versus 69.65 ± 19.82 N, respectively, for males and females.

Table 3 summarizes the relative differences in mBF between prosthetic groups, using Groups E (natural dentition), A (IFDP), and D (CRDP) as reference standards. These ratios are based on least squares mean values from a two‐way ANOVA, with statistical significance confirmed through Holm‐Šidák post hoc tests (*p* < 0.05).

**TABLE 3:** Relative ratios of maximum bite force across prosthetic groups using natural dentition (E), IFDP (A), and complete removable dentures (D) as reference standards

Group	LS mean (N)	Relative to E (natural dentition)	Relative to A (IFDP)	Relative to D (CRDP)
A—IFDP	523	0.69×	1.00 (ref)	7.26×
B—IOD (both arches)	294	0.39×	0.56×	4.08×
C—Max CRDP + Mand IOD	174	0.23×	0.33×	2.42×
D—CRDP	72	0.10×	0.14×	1.00 (ref)
E—Natural dentition	752	1.00 (ref)	1.44×	10.44×

*Note*: Relative ratios calculated using Group E (natural dentition), Group A (implant‐supported complete fixed denture—IFDP), and Group D (complete removable dental prosthesis—CRDP) as reference standards. Higher values indicate a closer approximation to natural dentition bite force.

**FIGURE 3:**
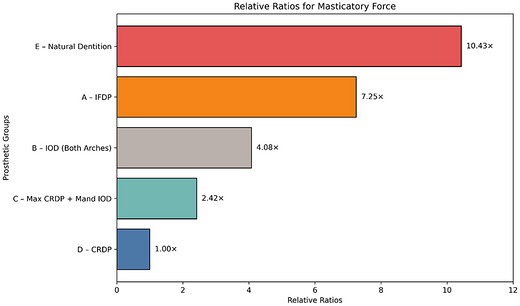
Relative ratios of maximum bite force (mBF) across prosthetic groups. Ratios are calculated using least squares means, with Group D (CRDP) as the reference standard. CRDP, complete removable dental prosthesis; IFDP, implant‐supported fixed dental prosthesis; IOD, implant‐retained overdenture.

Figure 3 illustrates the relative ratios in mBF across various dental prosthesis groups, using CRDP (Group D) as the reference standard. Each bar represents the relative mBF of a prosthetic condition compared to Group D, based on least squares means derived from two‐way ANOVA. Statistically significant differences (*p* < 0.05, Holm‐Šidák method) support the progressive increase in mBF from removable to implant‐supported and full natural dentition groups.

The repeated‐measures ANOVA revealed a highly significant difference among total, dominant, and nondominant mBF (F(2,334) = 136.30, *p* < 0.0001). Post hoc paired *t*‐tests confirmed significance for all pairwise comparisons (*p* < 0.0001). Mean values were 347.67 N (total), 224.89 N (dominant), and 190.85 N (nondominant), yielding stable ratios: total was ∼1.55× the dominant and 1.82× the nondominant, while the dominant was ∼1.18× stronger than the nondominant side.

Correlation analyses for mBF demonstrated that IFDPs were most similar to natural dentition (*r* = 0.60, *p* < 0.001), whereas CRDPs showed the greatest divergence (*r* = –0.65, *p* < 0.001). Strong bilateral correlations were observed between total and dominant side mBF (*r* = 0.93, *p* < 0.0001) and between total and nondominant side (*r* = 0.91, *p* < 0.0001). A near‐perfect correlation was also found between dominant and nondominant sides (*r* = 0.97, *p* < 0.0001), although variability in dominant‐to‐total ratios demonstrated that total mBF cannot be consistently estimated from unilateral values.

A two‐way ANOVA followed by Holm‐Šidák post hoc analysis revealed differences in speech intelligence scores among the prosthetic groups (*p* < 0.05). The full natural dentition group (E) demonstrated the highest mean score (85.67 ± 1.63 SEM), including the CRDP group (D; 49.96 ± 4.18 SEM, *p* < 0.001), the IODs in both arches group (B; 56.52 ± 3.36 SEM, *p* < 0.001), the maxillary CRDP with mandibular IOD group (C; 58.17 ± 3.34 SEM, *p* < 0.001), and the IFDP group (A; 70.13 ± 3.17 SEM, *p* = 0.0016).

Additionally, individuals with an IFDP (at least one arch) also showed a higher speech intelligence score compared to those wearing CRDP in both arches (Group D; *p* = 0.0002). No other intergroup differences were statistically significant. No statistically significant difference (*p* = 0.065) was observed between genders.

**FIGURE 4:**
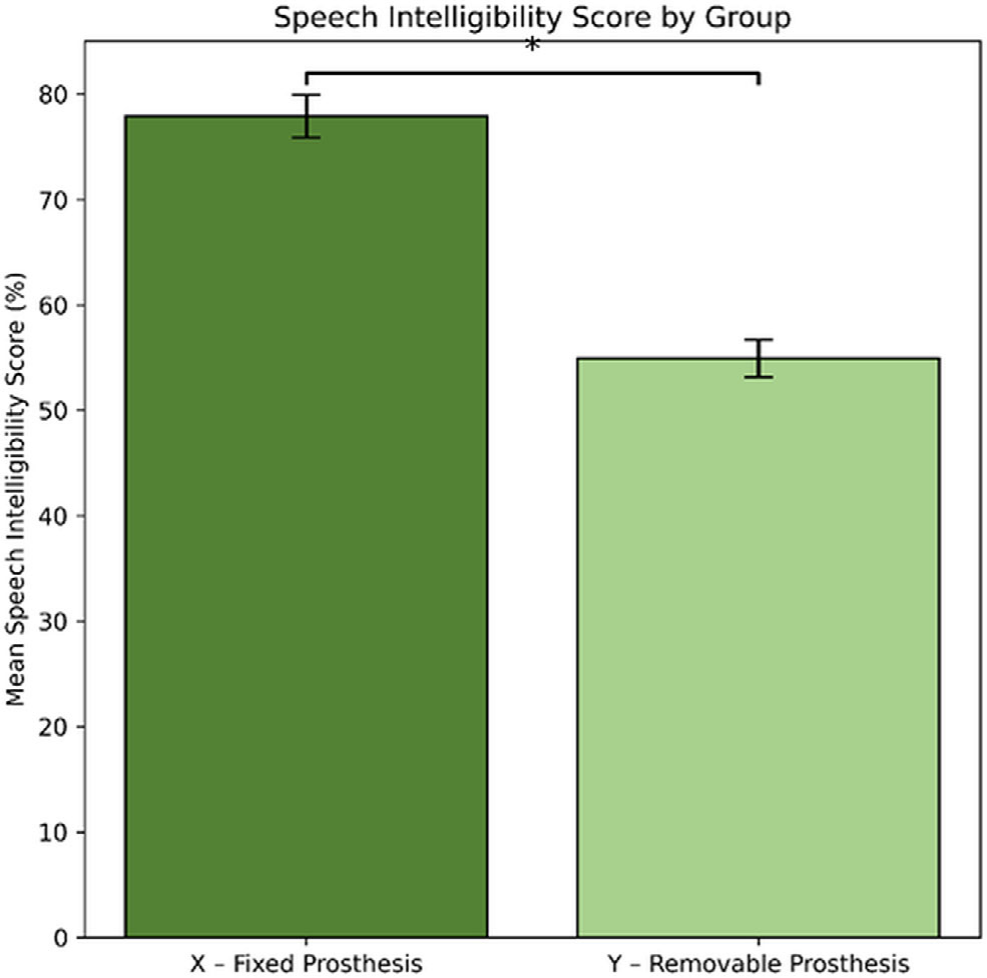
Speech intelligibility scores between fixed and removable prostheses. Mean values (±SEM) are displayed, comparing fixed prostheses (Groups A and E) with removable prostheses (Groups B–D).

A two‐way ANOVA was conducted to evaluate the effects of prosthesis type (fixed vs. removable) and gender on SI (Figure 4). The analysis revealed a significant main effect of prosthesis type (F(1,164) = 48.23, *p* < 0.001), with participants with natural dentition or receiving fixed prostheses (Group X) exhibiting higher scores than those with removable prostheses (Group Y). Additionally, the interaction between prosthesis type and gender was not significant (*p* = 0.369).

## DISCUSSION

For maximum bite force, statistically significant differences were observed across all groups. In contrast, for SI, statistical significance was observed between fixed and removable prosthetic designs, but not consistently across all individual prosthetic groups. Together, these findings demonstrate significant between‐group differences in both mBF and SI, thereby rejecting the null hypothesis for mBF and partially rejecting it for SI.

### Maximum bite force

This investigation provides a direct, objective comparison of functional outcomes across a full spectrum of contemporary prosthetic designs. To date, no study has comprehensively compared masticatory force across the full spectrum of prosthetic options. This study addressed this by evaluating complete dentures, implant‐retained overdentures (in one or both arches), implant‐supported fixed dental prosthesis, and natural dentition. The findings of this study establish a clear functional gradient for approaching maximum bite force.

### Natural dentition > IFDP > IOD > maxillary CRDP + mandibular IOD > CRDP

Fayad et al. documented functional gains over 6 months and emphasized the role of alveolar ridge morphology, duration of wear, gender, and denture material in influencing outcomes.^22^ The patterns in this study suggest that gender effects on mBF are most evident in groups with greater functional capacity. A previous study reported the influence of muscle thickness values on higher mBF in males.^21^ Similar gender‐related differences were observed, as males demonstrated significantly higher bite forces in the natural dentition and fixed restoration groups.

Denture wearers consistently exhibit reduced masticatory function, largely due to age‐related muscle atrophy and tooth loss.^23^ Restoration of function remains a central goal of prosthodontics, with removable and fixed prostheses offering different levels of rehabilitation.^11^ This study quantifies these differences in functional rehabilitation—maximum bite force with overdentures is approximately 39% and conventional dentures is 10% of dentate individuals.

CRDPs produced the lowest forces in this study. Kapur and Soman reported performance at approximately 30% of natural dentition based on masticatory efficiency assessed through food comminution testing, whereas this study demonstrated nearly a 90% reduction in maximum bite force relative to natural dentition.^24^ This apparent discrepancy reflects the distinct functional constructs being measured, as chewing efficiency describes the number of masticatory cycles required to pulverize food, while maximum bite force reflects the magnitude of force generated during a single occlusal contact and the capacity of an individual bite to fragment a food bolus. Addition of implants improved function substantially, with a 2.4‐fold increase in maximum bite force observed with two mandibular implants and a 4.08‐fold increase with implants in both arches. These results align with the McGill consensus advocating mandibular implant overdentures as the standard of care.^25^

While factors such as age, gender, prosthesis duration, muscular activity, and bone physiology may influence outcomes, this study highlights the independent contribution of prosthetic design. Rismanchian et al. reported improved bite force and satisfaction with complete dentures over time.^26^ The control group in this study was younger and not age‐matched to the prosthetic groups; because maximum bite force increases through adolescence and declines in older adults, and speech performance is likewise affected by age‐related neuromuscular changes, this difference may have influenced the magnitude of functional differences observed.^21^ Accordingly, limitations of this study include the single time‐point assessment, which does not account for the duration of prosthesis wear or potential adaptation. Larger, more standardized samples in future studies may further clarify the effects of demographic and physiologic variables.

Elysad and Mostafa demonstrated that telescopic partial dentures generated higher bite force than complete dentures, attributing this to improved retention and stability.^27^ Mericske‐Stern et al. showed that rigid bars enhance retention and vertical force distribution, effectively converting implant–tissue‐supported overdentures into implant‐supported designs.^28^ Bakke et al. confirmed improved biting and chewing efficiency after mandibular implant overdenture placement using electromyography in 12 patients.^29^ This study supports these findings by confirming that increased prosthesis retention is associated with higher maximum bite force, while additionally providing an objective comparison of functional outcomes across a full spectrum of contemporary prosthetic designs.

Fontijn‐Tekamp et al., in a larger cohort of 68 patients, found implant‐retained overdentures produced significantly higher unilateral and bilateral masticatory function than complete dentures, with no difference between implant‐borne and mucosa–implant‐borne systems.^9^ The MEM pressure sensor, Innobyte, provided reliable unilateral and bilateral measurements. Grouping unilateral values into dominant and nondominant sides revealed significant correlations across the dataset, confirming both the reproducibility of this device and the clinical utility of unilateral bite force assessment, particularly in conditions such as bruxism.^1^

Natural dentition produced the highest mBF in this study (median 744.5 N), closely aligning with values reported by Ustrell‐Barral et al. using the same MEM pressure sensor—Innobyte (mean 738.5 N). This concordance may support measurement consistency of the Innobyte system for maximum bite force assessment in prosthodontics.

### Speech intelligibility

Speech rehabilitation in prosthodontics has been less extensively studied than bite force. In this study, SI scores were significantly higher for natural dentition and implant‐supported fixed dental prostheses (IFDP) compared with denture groups. No gender differences were observed. These findings provide a foundation for future studies exploring long‐term adaptation, patient‐reported outcomes, and emerging technologies to further enhance chairside assessment and functional restoration.

Earlier investigations, including that of Lundqvist et al., evaluated speech outcomes by comparing phonetic characteristics within individuals before treatment with complete removable dentures and after rehabilitation with maxillary implant‐supported fixed prostheses.^15^ Tanaka et al. reported lower intelligibility in denture wearers compared with natural dentition, with improvement over time attributed to neuromuscular adaptation.^30^

Consistent with the findings of Tanaka et al., this study observed higher SI in fixed prostheses and natural dentition compared with removable designs. Differences from Lundqvist's observations may reflect variations in study methodology, including before‐and‐after treatment comparisons versus cross‐group analyses, as well as advances in implant and prosthesis design. Although phonetic principles are routinely incorporated during prosthodontic fabrication, objective post‐treatment assessment of SI across prosthetic designs remains limited, and this study provides quantitative cross‐group evidence supporting the influence of prosthetic design on speech outcomes.

## CONCLUSION

Within the scope of this study, maximum bite force and SI may serve as simple and reliable chairside measures for assessing functional performance across contemporary prosthodontic treatment options. These could serve as useful quantitative aids in evaluating the success of prosthetic procedures. The observed functional gradient offers a useful reference for patient education and treatment planning. Further research is needed to individually evaluate neuromuscular adaptation and to better delineate the influence of potential confounders, including age, gender, prosthesis wear duration, muscle activity, and bone physiology, on long‐term functional outcomes.

## CONFLICT OF INTEREST STATEMENT

The authors declare no conflicts of interest.
